# Visible-light-accelerated oxygen vacancy migration in strontium titanate

**DOI:** 10.1038/srep14576

**Published:** 2015-09-30

**Authors:** Y. Li, Y. Lei, B. G. Shen, J. R. Sun

**Affiliations:** 1 Beijing National Laboratory for Condensed Matter & Institute of Physics, Chinese Academy of Sciences, Beijing 100190, Peoples’ Republic of China

## Abstract

Strontium titanate is a model transition metal oxide that exhibits versatile properties of special interest for both fundamental and applied researches. There is evidence that most of the attractive properties of SrTiO_3_ are closely associated with oxygen vacancies. Tuning the kinetics of oxygen vacancies is then highly desired. Here we reported on a dramatic tuning of the electro-migration of oxygen vacancies by visible light illumination. It is found that, through depressing activation energy for vacancy diffusion, light illumination remarkably accelerates oxygen vacancies even at room temperature. This effect provides a feasible approach towards the modulation of the anionic processes. The principle proved here can be extended to other perovskite oxides, finding a wide application in oxide electronics.

Strontium titanate (SrTiO_3_, STO) is a model transition metal oxide, receiving intensive attentions due to its abundant physical properties. It is ordinarily paraelectric due to quantum fluctuation but undergoes a ferroelectric transition when stressed by lattice strains^1^,^2^,^3^ or electrical field^4^,^5^,^6^; it is typically insulating but exhibits a resistive transition to electronically conducting^7^, even to superconducting^8^ when it is oxygen-deficient. Due to its versatile properties, it serves as battery^9^, chemical sensors^10^, photo-catalysts^11^, and resistance-random-access-memory (RRAM) cell for non-volatile data storage^12^. Since most of these properties are closely related to the in- and ex-corporation reactions of oxygen, particularly the diffusion of oxygen vacancies (V_O_s), tuning the ionic processes is then highly desired. We noted that most of the previous work focused more on understanding than on tuning of the kinetic behaviors of V_O_s, and the interested issues include the forms of oxygen vacancies in STO (isolated V_O_, V_O_-pairs, or the V_O_ trapped by defect)^13^,^14^,^15^,^16^,^17^,^18^, their influence on anionic diffusion^19^,^20^, and spatial distribution of oxygen vacancies in STO^21^,^22^. Although there are attempts to modify the incorporation of oxygen^23^ or to generate oxygen defects on STO surface through ultraviolet light irridiation^24^, data in this regard are still very limited. The tuning of the kinetic behavior of V_O_s remains challenging, required further exploration. Here we reported on a dramatic tuning of the mobility of oxygen vacancies in STO by visible light illumination. It was found that, through exciting in-gap states of STO, light illumination depresses the activation energy for vacancy diffusion, remarkably accelerating the diffusion of V_O_s. Through speeding up ionic transport, this effect will find its applications in oxide electronics.

## Results

### Electrical field-induced and light illumination-accelerated structural deformation

**Figure 1:**
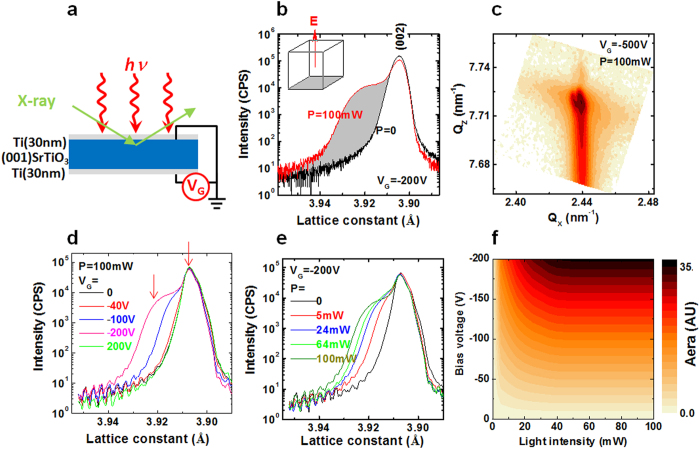
Effect of combined electrical and optical stimuli on the structure of STO. (**a)** A sketch of the experimental setup for x-ray diffraction. (**b)** XRD spectra of the 002 reflection of (001)-STO, recorded right upon the application of an electrical field along the [001]-axis. The duration of each ϑ–2ϑ scanning is 180 s. Shaded area marks the difference of the two XRD spectra with and without light illumination. The inset sketch shows the field direction with respect to the axes of the unit cell. (**c)** A reciprocal mapping of the 103 reflection of STO, measured under the conditions of *V*_G _= −500 V and *P = *100 mW. The downward tail of the main reflection marks the *c*-axis lattice expansion. (**d)** Structural changes for a constant light illumination but different gate fields. Positive gate bias produces no effect on structure even aided by illumination. Arrows mark the positions of the Bragg reflections. (**e)** Structural changes with light power while gate voltage is kept constant. (**f)** Distribution of structural deformation on *V*_G_-*P* plane, showing the combined effect of the electrical stressing and light illuminating. Light wavelength adopted here is λ = 532 nm. In all cases the leakage current is lower than 10 nA. All measurements were conducted at room temperature.

As schemed in Fig. 1a, samples are (001)-orientated STO substrates of the dimension of 5 × 5 × 0.5 mm^3^. As electrodes, two Ti layers were deposited respectively on the top and bottom surfaces through magnetron sputtering in an Ar atmosphere of 0.5 Pa. A gate voltage, *V*_G_, between −200 V and 200 V was applied to the back gate of STO while the top surface was grounded. In all cases, the leakage current is lower than 10 nA at the ambient temperature, which rules out the effect of Joule heating. Fig. 1b presents the x-ray diffraction (XRD) spectra of the 002 Bragg reflection of STO, recorded in the presence or absence of light. Without illumination, a bias voltage up to ±200 V produces negligible effects on the structure of STO. This can be ascribed to the low mobility of oxygen vacancies (~8 × 10^−12^ cm^2^/Vs at room temperature^20^).As recently reported, the structural deformation only occurs for STO accompanying the electro-migration of V_O_s^6^. It is easy to calculate the time required for the V_O_s to drift out of the interfacial layer, and it is ~6200 s under the gate bias of −200 V if layer thickness is ~2 μm, well beyond the time window of the ϑ–2ϑ scanning (~180 s). Aided by light, however, a gate bias of −200 V is high enough to cause sizable structural deformation. As shown in Fig. 1b, an obvious shoulder appears beside the main reflection when illuminated by a light of *P *= 100 mW (λ = 532 nm), indicating a lattice expansion along [001] axis. Since the unchanged phase can be clearly seen, the deformed phase could be much thinner than the penetration depth of x-ray in STO (~6 μm), appearing in an interfacial layer. According to reciprocal space mapping of the 103 reflection, fascinatingly, this new phase has an elongated *c-*axis lattice constant but a unchanged *a*-axis one (Fig. 1c). In contrast, positive biases produce no effect on lattice even aided by illumination, which means that the field-induced lattice expansion only appears underneath anode (Fig. 1d).

For a thorough exploration of this new gating effect, we measured the XRD spectra for various combinations of *V*_G_ and *P*. Fixing *P* to 100 mW while varying *V*_G_, according to Fig. 1d, lattice expansion emerges when *V*_G_ exceeds −40 V. From *V*_G _= −40 V to −200 V, the lattice constant changes from 3.905 Å to 3.922 Å (marked by red arrows), increasing by ~0.4%. While fixing *V*_G_ to −200 V but tuning *P*, sizable gating effect appears when *P *= 5 mW (Fig. 1e), i.e., the structural distortion caused by the gating effects can be accelerated remarkably even in low light power. Quantifying the lattice expansion in Fig. 1b by a shaded area, we obtain a quantitative description of the gating effects in the *V*_G_−*P* plane (Fig. 1f), clearly showing the combined effects of gate field and light illumination. As suggested in ref. 6, structural deformation occurs accompanying the electro-migration of V_O_s, these results suggest a mobilization of oxygen vacancies by light illuminating.

### Photo-excitation-modulated lattice expansion

**Figure 2:**
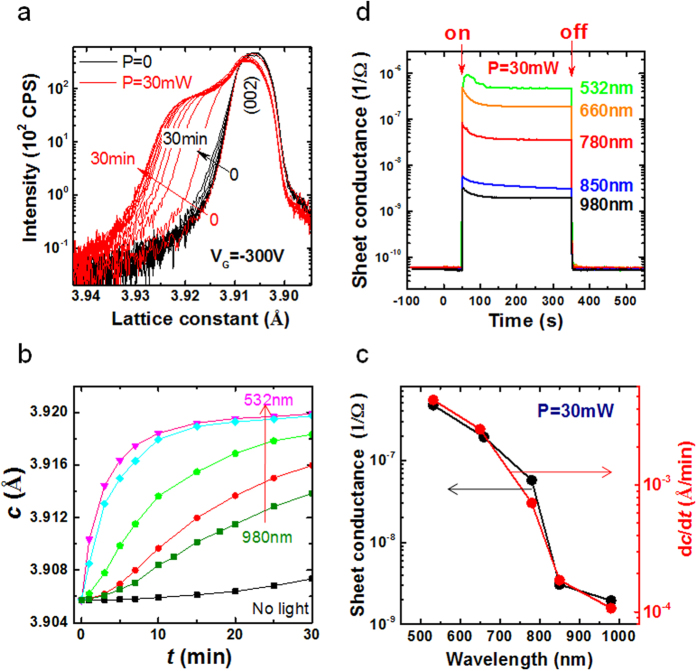
Concomitant variation in sheet conductance and structural deformation. (**a)** Evolution of the 002 reflection of STO with gating time, where *V*_G _= −300 V, *P *= 0 or 30 mW, and λ = 532 nm. (**b)** Lattice constant as a function of time, where *V*_G _= −300 V, *P *= 0 or 30 mW, and λ varies between 532 nm and 980 nm. (**c)** Sheet conductance of the LAO(3uc)/STO interface corresponding to the light ON/OFF operation, where *P *= 30 mW and λ varies between 532 nm and 980 nm. The applied current for resistive measurement is 1 μA. (**d)** A comparison of sheet conductance and structural change. Solid lines are guides for the eye. All measurements were performed at room temperature.

To reveal the effect of light illumination, the evolution of the 002 reflection with time is recorded when STO is exposed to different lights in the presence of electrical bias. Figure 2a shows the XRD spectra for two typical cases of *P = *0 and 30 mW (λ = 532 nm, *V*_G _= −300 V). Figure 2b is a summary of the data for different wavelengths (*P *= 30 mW). As shown in Fig. 2b, the acceleration of short wavelength light to gating effect is prompt: Lattice expansion occurs right upon the application of *V*_G_ and completes within about 10 minutes. Increasing λ leads to an inclining of the *c*(*t*) curve, indicating a slowdown of the gating effect. To get a quantitative description of the wavelength effect, in Fig. 2c we present, in semi-logarithmic scale, the dependence of *dc*/*dt* on λ, derived from the *c*(*t*) curve in the initial stage of structural deformation. A simple calculation shows that *dc*/*dt* is ~0.0047 Å/min for λ = 532 nm and ~0.0001 Å/min for λ = 980 nm, depressing nearly 50-fold.

The distinctive *dc*/*dt*−λ relation implies that the acceleration of the gating effect may be ascribed to photo-excitation rather than thermal activation. This is understandable since the photon energy used in the present experiments is well beyond the range of phonon energy. In fact, a direct effect of photo-excitation could be observed on photoconductivity. Since STO is insulating, its photoconductivity cannot be directly measured with visible light. We choose a LaAlO_3_/SrTiO_3_ interface with a 3-unit-cell-thick LaAlO_3_ top layer as a sample. As well established^25^, the interface will be insulating when the LaAlO_3_ layer is thinner than 4 unit cells for lack of mobile sheet charge carriers. However, we found that this insulating interface can be driven into conductive state by illuminating. As exemplified by Fig. 2d, from λ = 532 nm to 980 nm (*P *= 30 mw), the sheet conductance changes from *G *≈ 2 × 10^−5 ^Ω^−1^ to ~6 × 10^−8 ^Ω^−1^. It is obvious that light illumination generates extra charge carriers by exciting the in-gap states of STO (photon energy is lower than the band gap of STO), leading to the growth of sheet conductance. When the quantum efficiency for photo-excitation decreases with the increase of λ, *G* accordingly decreases. Surprisingly, the *G*-λ relation mimics the *dc*/*dt*-*λ* dependence very well, even in detailed features (Fig. 2c). This result strongly suggests that it is the photo-excitation of the in-gap states of STO that accelerates the gating effect. We noted that the light illumination induced conductance enhancement has been observed previously^26^,^27^. These works revealed the photo-excitation-generated effect. According to our data, we believed that photo-excitation of the in-gap state of STO, which gives rise to photoconductivity as shown in Fig. 2d, causes a transition of V_O_^•^ to V_O_^••^ + e, accelerating vacancy diffusion, as will be discussed later.

### Illumination-accelerated oxygen vacancies

**Figure 3:**
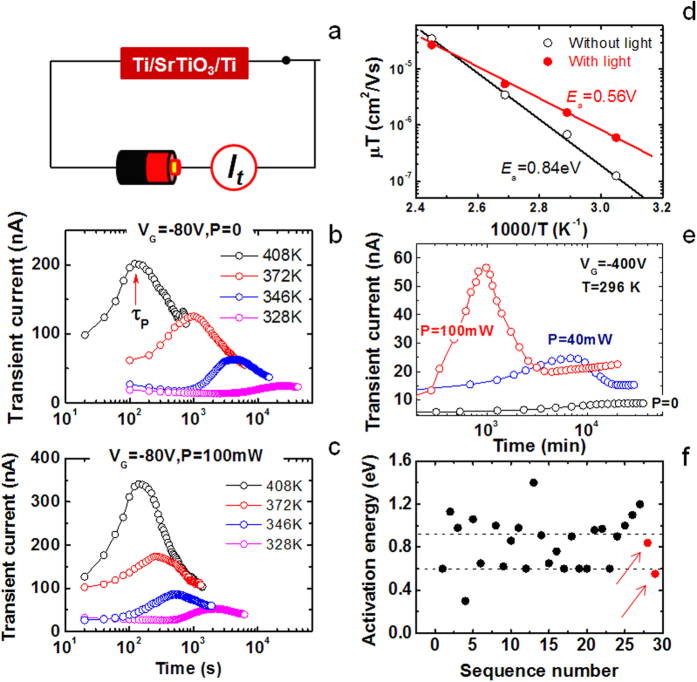
Transient current through biased STO. (**a)** A sketch of the experimental setup for transient current measurements. (**b)** Transient current for *V*_G _= −80 V and *P *= 0, measured under different temperatures. Arrow marks the position of current peak. (**c)** Transient current for *V*_G _= −80 V and *P *= 100 W (λ = 532 nm). (**d)** Semi-logarithmic plot of the vacancy mobility against reciprocal temperature. Solid lines are guides for the eye. (**e)** Transient current measured under different light powers (*V*_G _= −400 V, λ = 532 nm). (**f)** Activation energies of oxygen vacancies obtained by different groups (refs 18, 19, 20). Our results are represented by two red symbols (marked by two arrows). Dashed lines are guides for the eye.

A further question is how photo-excitation accelerates the gating effect. As suggested by Hanzig *et al.*^6^, the structural deformed phase forms while oxygen vacancies drift along electric field. In this scenario, photo-excitation may affect the gating effect through accelerating vacancy diffusion. To reveal the effect of light illumination on oxygen vacancies, transient current, *i*(*t*), through an electrically biased STO is studied in the presence (absence) of light illumination. Figure 3a is a schematic experimental setup. Figure 3b,c display the transient current as a function of time, recorded by fixing *V*_G_ to − 80 V at a temperature between 328 K and 408 K. With the increase of *t*, the transient current undergoes first a low to high and then a high to low transition, leaving a current peak at τ_p_. This feature is observed in all *i*(*t*) curves acquired at different temperatures. Comparing with the data without light, we found that light illumination causes an obvious left shift of current peak. This phenomenon is particularly evident when temperature is not very high. For example, at a temperature of 328 K, τ_p_ is ~2100 s in light but ~24970 s without light, i.e., the time required to reach the current peak has been reduced by more than one order of magnitude by light illumination. This result implies an acceleration of vacancy diffusion. As well established, the transient current is carried by V_O_s, and the transient current gets a maximum when the oxygen vacancies underneath anode arrive at cathode^6^,^28^.

As predicted by space-charge-limited (SCL) theory, the peak position of *i*(*t*) has a close relation to the mobility, μ, of oxygen vacancies following the equation^28^,^29^.
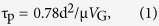


where *d* is the thickness of the sample. If the transient current is exclusively due to ionic charges, we will have the following equation
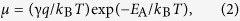


where *E*_A_ is the activation energy for oxygen vacancy diffusion, *k*_B_ is the Boltzmann’s constant, *T* is the temperature, *q* is the charge of oxygen vacancy, and γ is a pre-exponential factor. Here the Einstein relation between mobility and diffusivity, *μ *= *qD/k*_B_*T*, has been adopted for the derivation of Eq. (2). Based on Eq. (1), *μ* as a function of temperature can be deduced from the data in Fig. 3b,c. A direct calculation shows that the mobility is, for example, for *T *= 328 K, ~3.8 × 10^−10^ cm^2^/Vs without light and ~1.8 × 10^−9^ cm^2^/Vs with light. Extrapolating the data in Fig. 3b,c to room temperature (296 K), we obtain the vacancy mobility of ~1.8 × 10^−11^ cm^2^/Vs without light, which is comparable to the value deduced from ref. 20 (~8 × 10^−12^ cm^2^/Vs).

Remarkably, the *μ*-*T* relation is well described by Eq. (2). This, in addition to confirming the ionic origin of the transient current, reveals the activation nature of the charge transport process. From the slope of the semi-logarithmic *μT*-*T* plot in Fig. 3d, the activation energy can be deduced. It is ~0.84 eV without light and ~0.56 eV in a light of, for example, 100 mW and 532 nm. Light illumination reduces *E*_A_ by ~34%. These results indicate that, indeed, light illuminating mobilizes oxygen vacancies. Here we emphasize that the activation energy may be continuously depressed by illumination, as suggested by the progressive left shift of the current peak as *P* grows (Fig. 3e).

In fact, the diffusion of oxygen vacancies in STO has been intensively studied, and the activation energy obtained by different groups concentrated on two distinct categories around 0.6 eV and 1 eV (Fig. 3f)^19^,^20^. It is instructive to note that the activation energies deduced here (0.56 eV and 0.84 eV) also fall into these two groups (Fig. 3f). As will be discussed later, this may be an indication for illumination-induced changes in the V_O_-complexes that are believed to widely exist in STO, and this could be the underlying reason for the mobilization of V_O_s.

## Discussions

The above experiments suggest that light illumination accelerates field-induced lattice expansion via accelerating oxygen vacancy diffusion (Figs 1 and 3), and this acceleration process occurs accompanying the photo-excitation of in-gap states of STO (Fig. 2). A further question is how oxygen vacancy diffusion related to photo-excitation.

In general, it is believed that oxygen vacancies are doubly ionized at high temperatures, existing in the form of isolated V_O_^••^s^30^. According to Cordero^19^, the binding energy of a V_O_-complex is ~0.2 eV. A direct calculation shows that the probability for a V_O_-complex to be disassembled at 1000 °C is about 400-fold larger than that at 25 °C. At low temperatures, however, a singly ionized state could be favorable. By analyzing the relation between carrier concentration (*n*) and δ for SrTiO_3-δ_, Moos *et al.*^31^ declared that oxygen vacancies were singly ionized near room temperature, i.e., each V_O_ donates one charge carrier in the meantime trapping an electron. In their experiments, a well linear *n*−δ relation was observed from δ = 0.03 down to 0.01, without any signatures of inflection.

**Figure 4:**
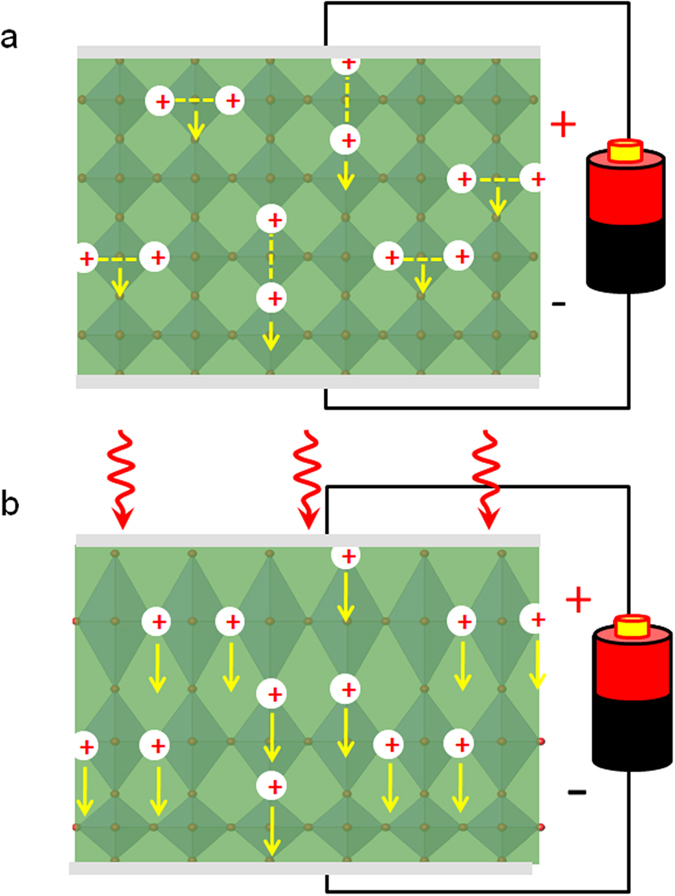
Schematic diagram for the migration of oxygen vacancies under electrical field and Light illumination. (**a)** Lattice deformation occurs accompanying the electro-migration of oxygen vacancies without light illumination. Oxygen vacancies near the STO surface may group into V_O_-complexes (for example, V_O_-Ti-V_O_ chains), and have a high activation energy for diffusion. As a consequence, the field-induced lattice change cannot be detected in the time window of the XRD experiments. (**b)** Photo-excitation of bounded electrons in the V_O_-complex leads to the disassembly of the V_O_-complexes, making oxygen vacancies much more mobile. Arrows mark the velocity of V_O_s in electrical field.

All of our experiments were conducted near room temperature. In this case, the content of singly ionized vacancies could be considerable. As well documented^15^,^16^,^19^,^30^, singly ionized vacancies prefer to form V_O_-complexes by sharing their electrons with the two closest titanium atoms, yielding in-gap states (Fig. 4a). In fact, V_O_-complexes in the form of Ti^3+^-V_O_ or V_O_-Ti-V_O_ pairs have been proposed^13^,^15^,^16^,^19^. Presumably, the *E*_A_ of these V_O_-complexes could be significantly larger than that of V_O_^••^s (ref. 9). Various experiments have shown that *E*_A_ is ~0.6 eV for V_O_^••^ and ~1 eV for V_O_-complexes^15^,^19^,^20^. Since the photon energy used here is much lower than the band gap of STO, photo-illumination will only affect the V_O_-complexes, exciting the shared electrons to the conduction band of STO. Without the shared electrons, however, the V_O_-complexes become unstable, disassembling into isolated V_O_^••^s (Fig. 4b). In this manner, photo-excitation accelerates the V_O_ diffusion thus the gating effect. This inference is in consistent with the observed illumination-induced reduction of *E*_A_ shown in Fig. 3d,f. To summarize, the present work reveals the close relation between photo-excitation and oxygen vacancy diffusion, paving the way towards the tuning of the anionic processes which could be important for oxide electronics.

## Methods

### Sample fabrication

Samples are (001)-orientated STO substrates (5 × 5 × 0.5 mm^3^) with an atomic level flat top surface. As electrodes, two 30-nm-thick Ti layers were deposited respectively on two surfaces through magnetron sputtering in an Ar atmosphere of 0.5 Pa. For comparison study, a LaAlO_3_/SrTiO_3_ sample with a LaAlO_3_ overlayer (3 unit cells in thickness) was prepared using the pulsed laser (248 nm) ablation technique. In the deposition process, the temperature was kept at 800 °C and the oxygen pressure at 10^−5^ mbar. The fluence of the laser pulses was 0.7 Jcm^−2^, and the repetition rate was 1 Hz. The layer thickness of LaAlO_3_ was *in situ* monitored by the RHEED (reflected high energy electron diffraction) technique. After deposition, the sample was annealed in 200 mbar of O_2_ at 600 °C for one hour, and then cooled to room temperature in the same oxygen pressure.

### Measurements

The structure of SrTiO_3_ was measured in the presence of a gate voltage and light illuminating, by a Bruker diffractometer (D8 Discover, Cu K_α_ radiation) with the x-ray being sparallelized and monochromatized by an asymmetric Ge 2202-Bounce monochromator. A transverse electrical field was applied to the bottom electrode of SrTiO_3_, while the top electrode was grounded. Lasers adopted in the present experiments have a wavelength between 532 nm and 980 nm. The spot size of the laser beam is 4 mm^2^, focusing on the regions where the x-ray was reflected. Ultrasonic Al wire bonding (20 μm in diameter) was employed for electrode connection for the LaAlO_3_/SrTiO_3_ sample. The four welding spots were well aligned, and the separation between neighbouring spots was ~0.4 mm. Laser beam focused on the area between two inner leads. The applied current for conductance measurements was 1 μA. All data were acquired at ambient temperature.

## Additional Information

**How to cite this article**: Li, Y. *et al.* Visible-light-accelerated oxygen vacancy migration in strontium titanate. *Sci. Rep.* **5**, 14576; doi: 10.1038/srep14576 (2015).

## References

[b1] Devonshire A. F. Theory of ferroelectrics. Phil. Mag. Suppl. 3, 85–130 (1954).

[b2] Haeni J. H. *et al.* Room-temperature ferroelectricity in strained SrTiO_3_. Nature 430, 758–761 (2004). 15306803 10.1038/nature02773

[b3] Zubko P., Catalan G., Buckley A., Welche P.R. L. & Scott J. F. Strain-gradient-induced polarization in SrTiO_3_ single crystals. Phys. Rev. Lett. 99, 167601 (2007). 17995293 10.1103/PhysRevLett.99.167601

[b4] Singh-Bhalla G. *et al.* Built-in and induced polarization across LaAlO_3_/SrTiO_3_ heterojunctions. Nat. Phys. 7, 80–86 (2011).

[b5] Rössle M. *et al.* Electric-field-induced polar order and localization of the confined electrons in LaAlO_3_/SrTiO_3_ heterostructures, Phys. Rev. Lett. 110, 136805 (2013). 23581357 10.1103/PhysRevLett.110.136805

[b6] Hanzig J. *et al.* Migration-induced field-stabilized polar phase in strontium titanate single crystals at room temperature. Phys. Rev. B 88, 024104 (2013).

[b7] Calvani P. *et al.* Observation of a midinfrared band in SrTiO_3-δ_. Phys. Rev. B 47, 8917 (1993). 10.1103/physrevb.47.8917 10004938

[b8] Schooley J. F., Hosier W. R. & Cohen M. L. Superconductivity in semiconducting SrTiO_3_. Phys. Rev. Lett. 12, 474–475 (1964).

[b9] Hanzig J. *et al.* Strontium titanate: An all-in-one rechargeable energy storage material. Journal of Power Source 267, 700–705(2014).

[b10] Menesklou W. *et al.* High temperature oxygen sensors based on doped SrTiO_3_. Sensors and Actuators B: Chemical 59, 184–189 (1999).

[b11] Walter M. G. *et al.* Solar water splitting cells. Chemical Reviews 110, 6446–6473 (2010). 21062097 10.1021/cr1002326

[b12] Waser R. & Aono M. Nanoionics-based resistive switching memories. Nat. Mater. 6, 833–840 (2007). 17972938 10.1038/nmat2023

[b13] Stashans A. & Vargas F. Periodic LUC study of F centers in cubic and tetragonal SrTiO_3_. Mater. Lett. 50, 145–148 (2001).

[b14] Ricci D., Bano G., Pacchioni G. & Illas F. Electronic structure of a neutral oxygen vacancy in SrTiO_3_. Phys. Rev. B 68, 224105 (2003).

[b15] Cuong D. D. *et al.* Oxygen vacancy clustering and electron localization in oxygen-deficient SrTiO_3_: LDA + U study. Phys. Rev. Lett. 98, 115503 (2007). 17501064 10.1103/PhysRevLett.98.115503

[b16] Lin C. W. & Demkov A. A. Electron correlation in oxygen vacancy in SrTiO_3_. Phys. Rev. Lett. 111, 217601 (2013). 24313525 10.1103/PhysRevLett.111.217601

[b17] Muller D. A. *et al.* Atomic-scale imaging of nanoengineered oxygen vacancy profiles in SrTiO_3_. Nature 430, 657–661 (2004). 15295595 10.1038/nature02756

[b18] Wang X. *et al.* Static and ultrafast dynamics of defects of SrTiO_3_ in LaAlO_3_/SrTiO_3_ heterostructures. Appl. Phys. Lett. 98, 081916 (2011).

[b19] Cordero F. Hopping and clustering of oxygen vacancies in SrTiO_3_ by anelastic relaxation. Phys. Rev. B 76, 172106 (2007).

[b20] Souza R. A. D., Metlenko V., Park D. & Weirich T. E. Behavior of oxygen vacancies in single-crystal SrTiO_3_: Equilibrium distribution and diffusion kinetics. Phys. Rev. B 85, 174109 (2012).

[b21] Szot K, Speier W., Carius R., Zastrow U. & Beyer W. Localized metallic conductivity and self-healing during thermal reduction of SrTiO_3_. Phys. Rev. Lett. 88, 075508 (2002). 11863913 10.1103/PhysRevLett.88.075508

[b22] Liu Z. Q. *et al.* Metal-insulator transition in SrTiO_3-*x*_thin films induced by frozen-out carriers, Phys. Rev. Lett. 107, 146802 (2011). 22112172 10.1103/PhysRevLett.107.146802

[b23] Merkle R., Souza R. A. D. & Maier J. Optically tuning the rate of stoichiometry changes: surface-controlled oxygen incorporation into oxides under UV irradiation. Angew. Chem. 113, 2184–2187 (2001). 11433466

[b24] Mochizuki S., Fujishiro F. & Minami S. Photoluminescence and reversible photo-induced spectral change of SrTiO_3_. J. Phys.: Condens. Mater 17, 923–948 (2005).

[b25] Thiel S., Hammerl G., Schmehl A., Schneider C. W. & Mannhart J. Tunable quasi-two-dimensional electron gases in oxide heterostructures. Science 313, 1942–1945 (2006). 16931719 10.1126/science.1131091

[b26] Lu H. L. *et al.* Photoelectrical properties of insulating LaAlO_3_–SrTiO_3_ interfaces, Nanoscale 6, 736–740 (2014). 24301882 10.1039/c3nr05162e

[b27] Lu H. L. *et al.* Reversible insulator-metal transition of LaAlO_3_/SrTiO_3_ interface for nonvolatile memory, Scientific Reports 3, 2870 (2013). 24100438 10.1038/srep02870 PMC3792417

[b28] Zafar S. *et al.* Oxygen vacancy mobility determined from current measurements in thin Ba_0.5_Sr_0.5_TiO_3_ films. Appl. Phys. Lett. 73, 175–177 (1998).

[b29] Many A. & Rakavy G. Theory of transient space-charge-limited currents in solids in presence of trapping. Phys. Rev. 26, 1989 (1962).

[b30] Moos R. & Härdtl K. H. Defect chemistry of donor-doped and undoped strontium titanate ceramics between 1000°C and 1400°C. J. Am Ceram. Soc. 80, 2549–2562 (1997).

[b31] Moos R., Menesklou W. & Hardtl K. H. Hall mobility of undoped n-type conducting strontium titanate single crystals between 19 K and 1373 K. Appl. Phys. A 61, 389–395 (1995).

